# Brief Reports Prepared by Students and Graduates of Master of Science in Biobanking

**DOI:** 10.1089/bio.2022.0064

**Published:** 2023-02-15

## Introduction: *Biopreservation and Biobanking*—Short Communications of Graduates and Students of Master of Science in Biobanking in Europe

Karine Sargsyan^i^

International Biobanking and Education, Medical University of Graz, Graz, Austria.

I am inspired to introduce the readers of *Biopreservation and Biobanking* to a set of short communications describing several thesis projects conducted by the graduates and students of master of science in biobanking at the Medical University of Graz, Austria and French Master in Biobanks & Complex Data Management of Université Côte d'Azur in France (Table 1).

**Table 1.** List of Thesis Projects

	Project title and authors
1^a^	“Universal DataBase for the Geneva pediatric onco-hematology biobank—BaHOP.” Denis Marino and Karine Sargsyan.
2^a^	“ISO 9001:2015 versus ISO 20387:2018: which makes the difference for a biobank?” Ichata Boina, Caroline Dos Santos Rocha Maia, Nicolas Ferry, Laurine Mathieu, Beheshta Paiman, Iram Riaz, Darya Ryzhenkova, Paul Hofman, and Nicole Arrighi.
3^a^	“A snapshot on the regulatory activity of the Human Tissue Authority in the United Kingdom research sector.” Jorgelina Trueba and Berthold Huppertz.
4	“Moving towards personalized medicine with oncological biobanks: University-based biobanking model of international multicenter cooperation.” Anna Michalska-Falkowska and Karine Sargsyan.
5	“Standardization in Biobanking: Guide to implement ISO 20387:2018 in biobanks certificated with ISO 9001:2015.” Michael Zúñiga and Karine Sargsyan.
6	“Benchmarking study of animal biobanks in Europe.” Mbayame Diop, Paolo Bonvicini, Samantha Luciano, Tommy-Lee Banlier, Youmna Chelbi, Paul Hofman and Nicole Arrighi.
7	“Environmental Sustainability in Biobanking.” Anita Litschauer and Berthold Huppertz.
8	“Raising the awareness of biobanking in the Swiss population through higher education.” Nesa Marti and Karine Sargsyan
9	“Implementation of electronic informed consent for cancer research.” Nina Bertheussen Krüger and Karine Sargsyan.

^a^ These three concepts were chosen to be published in this print edition of *Biopreservation and Biobanking*. An additional six concepts have been published online as “22-0064.R1 6 online JV review” in Supplementary Data.

As is widely recognized, global developments in the multidisciplinary field of biobanking are intimately linked with the increasing demand for highly specialized biobanking experts. To address these needs, education in such a dynamic area should be provided by professionals responsible for translating the knowledge and experience into applied biobanking.

Exceptional opportunities for integrated and comprehensive studies in biobanking have been created at the Medical University of Graz in Austria (with a focus on the management aspects of biobanking) and the Université Côte d'Azur in France (with a focus on complex data usage in research).

The lecturers are dedicated to transferring their knowledge and first‐hand experience with practical implementation and management of biobanks at these two internationally recognized institutions to educate the next generations of biobankers. Within the extensive range of courses already offered by the Medical University of Graz, Bologna Convention conforming, the international master of science in biobanking postgraduate course covers all aspects of this interdisciplinary field. During the two years of studies, participants gain knowledge and practical support regarding the formation and management of national and international biobanks, including quality management, risk assessment, sustainability, budgeting, and cooperation with academic and industrial partners.

To make the course available to participants from the broadest range of countries around the world, all lecture exercises, online material, and interactive presentations are provided in English. Therefore, it is possible to actively attend the master's course and work full time in a desired part of the world. Multiple previously inaccessible opportunities await these master course graduates, after gaining broad knowledge and expertise in biobanking. This is evident from the many success stories of past graduates who describe promotions from technician to biobank manager or biobanking leader.

Working with students of the MSc in biobanking is inspiring. Our graduates, now developing successful and sustainable biobanks, cooperating within networks and with international partners, represent the best confirmation of benefits that came with the education and practice.

**Supplementary Material**

Supplementary Data

Address correspondence to:

*Karine Sargsyan, MD*

*International Biobanking and Education*

*Medical University of Graz*

*Graz*

*Austria*

*E‐mail:* karine.sargsyan@medunigraz.at

## Universal DataBase for the Geneva Pediatric Onco‐Hematology Biobank: BaHOP

Denis Marino^1,i^ and Karine Sargsyan^2,3,i,ii^

^1^Research Platform for Pediatric Oncology and Hematology, Department of Pediatrics, Gynecology, and Obstetrics, University of Geneva, Geneva, Switzerland.

^2^Department of International Biobanking and Education, Medical University of Graz, Graz, Austria.

^3^Department of Medical Genetics, Yerevan State Medical University, Yerevan, Armenia.

**Keywords:** biobank information management systems, BIMS requirements, IT systems for research human biobank, biobank workflow analysis

**Introduction**

Biobanks have gained importance for the development of sustainable research by generating high‐quality resources with clinical annotation of relevant biological samples.

The importance of the implementation and use of an IT management system for biobanks is acknowledged worldwide. Biobanks that are still using spreadsheets, paper records, and handwritten identifiers on storage tubes will be at a great disadvantage. Moreover, an IT management system can increase the quality of the biological resources stored in the repository and so *de facto* can contribute to increasing quality of research results. This in turn can also contribute to the reduction of irreproducible preclinical research, which according to Freedman et al^1^ is responsible for >60% of the total causes of irreproducibility.

Both the International Society for Biological and Environmental Repositories Best Practices and International Agency for Research on Cancer Common Minimum Technical Standards and Protocols for Biobanks Dedicated to Cancer Research^2,3^ dedicate special attention to the use of an effective biobank IT management solution that ensures effective tracking of biological resources during their entire lifecycle. Paskal et al and Bendou et al^4,5^ define the usage of an IT management system.

The goal of this master's thesis concept is to analyze the type and frequency of deficiencies in biobanks seeking licensing from a national licensing body.

**Methods**

Biobank workflow analysis and requirement identification

Several iterative cycles were performed for the analysis of the biobank workflow. A literature search on Biobank Information Management System (BIMS) implementation concepts and requirements was performed, followed by a further examination of selected publications^6–11^ and standards.^2,3,12–17^ Brainstorming techniques and unstructured interviews with biobank stakeholders were completed to categorize requirements related to laboratory activities.

Selection process for the identification of suitable software

The identification of adapted commercially available IT solutions was made in collaboration with the national biobank network, the Swiss Biobanking Platform. A first assessment was performed analyzing the number of features, summarized by Swiss universities under their data life‐cycle management project.^18^

In a second step, demonstration use cases were identified and provided to vendors. Previously identified requirements were used as a checklist to evaluate the performance and suitability. A selection of attributes and their ponderation scores was arbitrarily selected according to Geneva Pediatric biobank for research in onco‐hematology (BaHOP) specifications (Fig. 1).

**FIG. 1.**
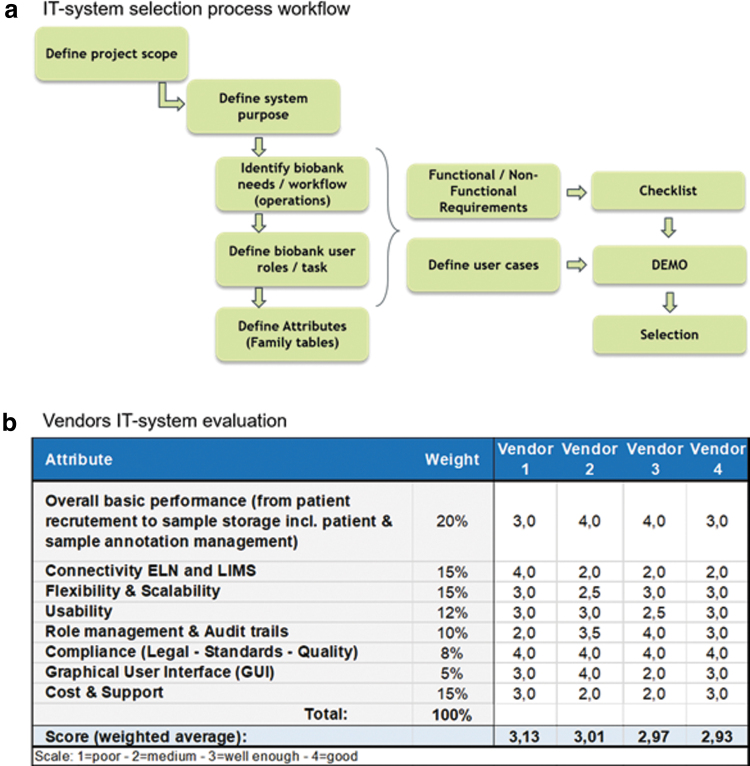
IT systems selection process and evaluation. **(a)** Shows the steps performed for the selection of the IT system. **(b)** Shows the results achieved by the different IT systems evaluated.

**Results**

Biobank workflow analysis and requirement identification

The workflow analysis enabled us to identify 13 main process categories and 123 requirements, which were grouped into 15 clusters and separated into 3 distinct categories: (1) functional requirements, (2) nonfunctional requirements, and (3) system operation.

BIMS selection and implementation process

We observed a notable difference for some criteria, as well as unanimity in ratings for legal compliance, standards, and quality recommendations. All vendors had good to excellent performance in executing the previously released script based on sample management (Fig. 1).

**FIG. 1.**
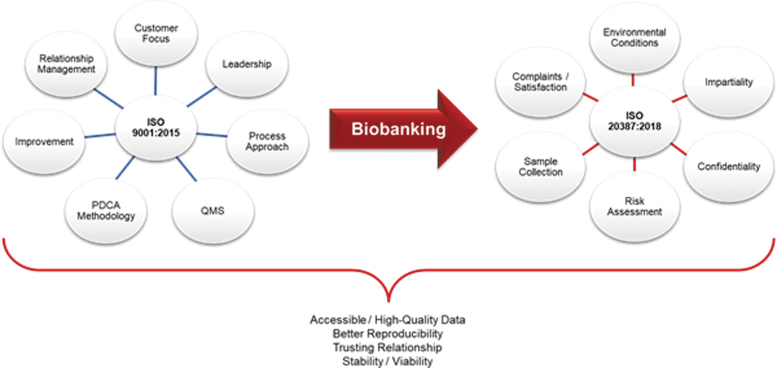
Main pillars supported by ISO 9001:2015 and ISO 20387:2018 norms, highlighting the differences between the two standards and the contribution of the implementation of ISO 20387:2018 for biobanks.

**Discussion**

Nonprofit small size academic research biobanks are widely developed by scientists for the need of research, with limited IT project management knowledge. This study corroborates Prokosch et al's^7^ results demonstrating the complexity of selection and implementation processes of a biobank information management system. Likely, BaHOP is similar to most of the biobanks not having specific resources allocated to IT project development as highlighted by Müller et al,^19^ where a survey in France showed that 80% of biobanks do not have resources for IT project development.

Furthermore, both authors underline that the selection of a dedicated BIMS is closely related to a series of critical attributes for which analysis results will diverge for every repository: (1) user license cost, (2) ability for purpose, (3) implementation period, and (4) constant support and maintenance. The selection of commercial systems has proven to be a required step toward quality and national process harmonization. We demonstrate and gave hints on how a small size research biobank can acquire an IT system by applying project management techniques.

**Acknowledgments**

We thank the team of the CANSEARCH research platform in pediatric oncology and hematology of the University of Geneva (Mary Khoshbeen‐Boudal, Laurence Lesne, Yoann Sarmiento), the Onco‐Hematology Unit of the HUG (Marc Ansari, Tiago Nava, Veneranda Mattiello, Rodolfo Lo Piccolo, Fanny Muet), and the Swiss Biobanking Platform.

**Authors' Contributions**

D.M. contributed to conceptualization, design, methodology, formal analysis, data curation, project administration, writing—all stages, and visualization. K.S. was involved in supervision, conceptualization, design, methodology, reviewing, and editing. All authors have approved the submitted version and have agreed both to be personally accountable for the author's own contributions and to ensure that questions related to the accuracy or integrity of any part of the study, even those in which the author was not personally involved, are appropriately investigated, resolved, and the resolution documented in the literature.

Ethics approval and consent to participate

The Geneva Cantonal Commission for Research Ethics has approved the BaHOP biobank (approval PB_2017‐00533)

**Author Disclosure Statement**

No conflicting financial interests exist.

**Funding Information**

This study is supported by the CANSEARCH Foundation. Further funding comes from the Swiss National Science Foundation (31BL30_185396). The funding bodies have no role in the design of the study and collection, analysis, and interpretation of data and in writing this article.

**References**

1. Freedman LP, Cockburn IM, Simcoe TS. The economics of reproducibility in preclinical research. PLoS Biol 2015;13(6):e1002165; doi: 10.1371/journal.pbio.1002165

2. Campbell LD, Astrin JJ, DeSouza Y, et al. The 2018 revision of the ISBER best practices: Summary of changes and the editorial team's development process. Biopreserv Biobank 2018;16(1):3–6; doi: 10.1089/bio.2018.0001

3. Mendy M, Caboux E, Lawlor RT, Wright J, Wild and CP. Common Minimum Technical Standards and Protocols for Biobanks Dedicated to Cancer Research. 2017. Available from: http://publications.iarc.fr/Book-And-Report-Series/Iarc-Technical-Publications/Common-Minimum-Technical-Standards-And-Protocols-For-Biobanks-Dedicated-To-Cancer-Research-2017 [Last accessed September 25, 2019].

4. Paskal W, Paskal AM, Dębski T, Gryziak M, Jaworowski J. Aspects of modern biobank activity—Comprehensive review. Pathol Oncol Res 2018;24(4):771–785; doi: 10.1007/s12253‐018‐0418‐4

5. Bendou H, Sizani L, Reid T, et al. Baobab laboratory information management system: Development of an open‐source laboratory information management system for biobanking. Biopreserv Biobank 2017;15(2):116–120; doi: 10.1089/bio.2017.0014

6. Quinlan PR, Mistry G, Bullbeck H, Carter A. A data standard for sourcing fit‐for‐purpose biological samples in an integrated virtual network of biobanks. Biopreserv Biobank 2014;12(3):184–191; doi: 10.1089/bio.2013.0089

7. Prokosch HU, Beck A, Ganslandt T, et al. IT infrastructure components for biobanking. Appl Clin Inform 2010;1(4):419–429; doi: 10.4338/ACI‐2010‐05‐RA‐0034

8. Nussbeck SY, Skrowny D, O'Donoghue S, Schulze TG, Helbing K. How to design biospecimen identifiers and integrate relevant functionalities into your biospecimen management system. Biopreserv Biobank 2014;12(3):199–205; doi: 10.1089/bio.2013.0085

9. Eminaga O, Semjonow A, Oezguer E, et al. An electronic specimen collection protocol schema (eSCPS). Methods Inf Med 2014;53(1):29–38; doi: 10.3414/ME13‐01‐0035

10. Späth MB, Grimson J. Applying the archetype approach to the database of a biobank information management system. Int J Med Inf 2011;80(3):205–226; doi: 10.1016/j.ijmedinf.2010.11.002

11. Schwanke J, Rienhoff O, Schulze TG, Nussbeck SY. Suitability of customer relationship management systems for the management of study participants in biomedical research. Methods Inf Med 2013;52(4):340–350; doi: 10.3414/ME12‐02‐0012

12. Betsou F, Lehmann S, Ashton G, et al. Standard preanalytical coding for biospecimens: Defining the sample PREanalytical code. Cancer Epidemiol Prev Biomark 2010;19(4):1004–1011; doi: 10.1158/1055‐9965.EPI‐09‐1268

13. Lehmann S, Guadagni F, Moore H, et al. Standard preanalytical coding for biospecimens: Review and implementation of the sample PREanalytical code (SPREC). Biopreserv Biobank 2012;10(4):366–374; doi: 10.1089/bio.2012.0012

14. Norlin L, Fransson MN, Eriksson M, et al. A minimum data set for sharing biobank samples, information, and data: MIABIS. Biopreserv Biobank 2012;10(4):343–348; doi: 10.1089/bio.2012.0003

15. BIMS guidelines—Swiss Biobanking Platform. Available from: https://swissbiobanking.ch/specification‐for‐a‐bims/ [Last accessed: May 26, 2020].

16. Data Collection. EBMT. Available from: https://www.ebmt.org/registry/data-collection [Last accessed: May 26, 2020].

17. Vocabularies for Biobanking | BBRB. Available from: https://biospecimens.cancer.gov/resources/vocabularies.asp [Last accessed: May 26, 2020].

18. ELN and LIMS:: DLCM. Available from: https://www.dlcm.ch/services/dlcm-eln [Last accessed: July 2, 2019].

19. Müller H, Malservet N, Quinlan P, et al. From the evaluation of existing solutions to an all‐inclusive package for biobanks. Health Technol 2017;7(1):89–95; doi: 10.1007/s12553‐016‐0175‐x

Address correspondence to:

*Karine Sargsyan, MD*

*Department of International Biobanking and Education*

*Medical University of Graz*

*Graz*

*Austria*

*E‐mail:* karine.sargsyan@medunigraz.at

## ISO 9001:2015 Versus ISO 20387:2018: Which Makes the Difference for a Biobank?

Ichata Boina,^1^ Caroline Dos Santos Rocha Maia,^1^ Nicolas Ferry,^1^ Laurine Mathieu,^1^ Beheshta Paiman,^1^ Iram Riaz,^1^ Darya Ryzhenkova,^1^ Paul Hofman,^1,2^ and Nicole Arrighi^1,3^

^1^MSc Biobanks and Complex Data Management, University Côte d'Azur, Nice, France.

^2^Biobank 0033‐00025 and FHU OncoAge, Nice Hospital Center, University Côte d'Azur, Nice, France.

^3^INSERM U1065, Centre Méditerranéen de Médecine Moléculaire, Université Côte d'Azur, Nice, France.

**Keywords:** biobanking standardization, biobank quality control, biobank sustainability

**Introduction**

Biobanks need to meet increasingly higher quality standards required by the scientific community and industry partners to ensure that the samples and clinical–biological associated data allow for high accuracy, comparability, and reproducibility.^1^ In 2015, Freedman et al estimated the reproducibility costs in preclinical research in the United States of America. About US$28B was spent without leading to reproducible results, mainly due to inappropriate handling of the specimen in the preanalytical phase.^2^

To improve reproducibility, standard guidelines used for biomedical research are currently being analyzed in biobanking processes. Biobanks that follow worldwide recognized guidelines are mainly certified compliant with the 9001:2015 standard of the International Organization for Standardization (ISO) but also according to other programs in North of America or to the S96‐900 norm in France.^3^ Although it is an adequate standard for the Quality Management System (QMS), biobanks are currently being encouraged to face the challenge of progressing from ISO 9001:2015 certification to ISO 20387:2018 accreditation.^4^

The goal of this master's thesis concept is to explore what difference the progression to ISO 20387:2018 may make for biobanking.

**Methods**

Define the scope of the ISO 9001:2015 standard

ISO 9001:2015 was published in 1987, adapted, then updated in September 2015. It is a quality management guideline that can be used by any business that provides a product or a service. It is based on seven main principles: customer focus, leadership, engagement of people, process approach, improvement, evidence‐based decision making, and relationship management.^5^ This guideline is mainly used to facilitate the development and improve the QMS.^6^ Furthermore, certification is provided by the regulatory body or an external company after an audit demonstrating abilities to produce goods or services.^7^

Identify the specificities of the ISO 20387:2018 standard

The ISO 20387:2018 general requirements for biobanking is a recent international standard, published in 2018, aiming to improve the confidence in biobank organizations within a third‐party covenant including donors, researchers, and regulatory bodies. It applies to all organizations performing biobanking, from multicellular organisms (e.g., humans, animals, and plants) to microorganisms, for research and development.^8^

It covers general specifications for impartiality and confidentiality, staff members' training and competence assessment, as well as consistent operation of biobanks, including higher levels of traceability and quality control to ensure biological material and data of adequate quality. The requirements are related to the biobank's missions, its well‐documented procedures and processes, and compliance with ethical and biosafety requirements.^8^

**Results**

Similarities and differences between ISO 9001:2015 and ISO 20387:2018

Many similarities can be found in both standards. They emphasize the importance of incorporating risk management into the design and production stages and developing strategies to minimize the negative effects of risk.

The main difference between the two ISOs is the scope and their application domain. ISO 9001:2015 is applicable to any organization, whereas ISO 20387:2018 deals specifically with those institutions performing biobanking. QMS performance will monitor quality objectives in sample processing. In research related to the health domain, associated clinical data issued from collected samples and corresponding medical records must be confidential. This security of personal data is crucial. Patients trust the research investigator, who will trust the biobank manager. With implementing ISO 20387:2018, the biobanks will deal with data confidentiality, patient satisfaction, and legal and ethical issues (Fig. 1).

The importance of implementing ISO 20387:2018: sustainability

Sustainability is a pivotal point of a biobank, and ISO 20387:2018 requires the biobank to have a strategy concerning financial viability, which must be reviewed periodically.^9,10^ This dynamic strategy will assist the biobank in long‐term project planning.

To obtain the accreditation, the biobank needs to invest around €15,000 to €25,000 for the whole process and between €8,000 and €10,000 to maintain the accreditation for a duration of 4 years.

**Discussion**

The first ISO 20387:2018 accredited biobank in the world was the Cornell Veterinary Biobank.^11^ ISO 20387:2018 accreditation reflected the high level of the biobank quality management and processes and permitted the structure to receive a $2.5 million national grant for a dog aging project.^12^ This illustrates the opportunity given to biobanks when they comply with this specific norm, the stability gains, and the recognition by international instances that may facilitate projects and collaborations.

We can consider within the impact of the implementation of the ISO 20387:2018 the greater access and quality of data, which supports improved reproducibility of scientific studies, and the improved trust of donors, researchers, and regulatory bodies. Besides, ISO 20387:2018 requiring a strategy concerning financial viability will lead to an impact on the biobank community. There are currently more and more biobanks in the world, and most of them already have some form of QMS. But with the adoption of this norm, most of them may be more stable and viable through the years.

**Authors' Contributions**

I.B. and C.D.S.R.M. contributed to writing, reviewing, and editing. N.F. carried out writing, figure design, and reviewing. L.M. carried out writing, reviewing, and editing. B.P., I.R., and D.R. were involved in writing. P.H. and N.A. were in charge of supervision.

**Author Disclosure Statement**

No conflicting financial interests exist.

**Funding Information**

No funding was received.

**References**

1. De Blasio P, Biunno I. New challenges for biobanks: Accreditation to the new ISO 20387:2018 standard specific for biobanks. BioTech 2021;10(3):13; doi: 10.3390/biotech10030013

2. Freedman LP, Cockburn IM, Simcoe TS. The economics of reproducibility in preclinical research. PLoS Biol 2015;13(6):e1002165. doi: 10.1371/journal.pbio.1002165

3. Barnes RO, Shea KE, Watson PH. The Canadian tissue repository network biobank certification and the College of American pathologists biorepository accreditation programs: Two strategies for knowledge dissemination in biobanking. Biopreserv Biobank. 2017;15(1):9–16. doi: 10.1089/bio.2016.0021

4. Betsou F. Quality assurance and quality control in biobanking. In: Biobanking of Human Biospecimens: Principles and Practice (Hainaut P, Vaught J, Zatloukal K, Pasterk M, eds.). Springer International Publishing: Cham, Switzerland; 2017; pp. 23–49.

5. ISO. ISO 9001:2015—Quality management systems—Requirements. 2015. Available from: https://www.iso.org/standard/62085.html consulted [Last accessed: January 24, 2022].

6. Grizzle WE, Gunter EW, Sexton KC, Bell WC. Quality management of biorepositories. *Biopreserv Biobank* 2015;13(3):183–94. doi: 10.1089/bio.2014.0105

7. Gaignaux A, Ashton G, Coppola D, et al. A biospecimen proficiency testing program for biobank accreditation: Four years of experience. *Biopreserv Biobank* 2016;14(5):429–439; doi: 10.1089/bio.2015.0108

8. ISO. ISO 20387:2018—BiotechnologyBiobanking—General requirements for biobanking. 2018. Available from: https://www.iso.org/standard/67888.html [January 24, 2022].

9. Odeh H, Miranda L, Rao A, et al. The biobank economic modeling tool (BEMT): Online financial planning to facilitate biobank sustainability. *Biopreserv Biobank* 2015;13(6):421–429. doi: 10.1371/journal.pbio.1002165

10. Klingler C, von Jagwitz‐Biegnitz M, Baber R, et al. Stakeholder engagement to ensure the sustainability of biobanks: A survey of potential users of biobank services. Eur J Hum Genet (2021); doi: 10.1038/s41431‐021‐00905‐x

11. Mouttham L, Garrison SJ, Archer DL, Castelhano MG. A biobank's journey: Implementation of a quality management system and accreditation to ISO 20387. Biopreserv Biobank 2021;19(3):163–170; doi: 10.1089/bio.2020.0068

12. Ramanujan K. Grant launches dog aging project biobank at Cornell [Internet]. Cornell Chronicle 2020. Available from: https://news.cornell.edu/stories/2020/07/grant-launches-dog-aging-project- [Last accessed November 29, 2021].

*Nicole* *Arrighi, PhD*

*MSc Biobanks and Complex Data Management*

*University Côte d'Azur*

*Nice*

*France*

*E‐mail:* nicole.arrighi@univ‐cotedazur.fr

## A Snapshot on the Regulatory Activity of the Human Tissue Authority in the United Kingdom Research Sector

Jorgelina Trueba^1,i^ and Berthold Huppertz^2,i^

^1^Cancer Research UK, Cambridge Institute, University of Cambridge, United Kingdom.

^2^Head of Gottfried Schatz Research Center, Medical University of Graz, Graz, Austria.

**Keywords:** biobanking, ethics in biobanking, staff at biobank, quality standard at biobank

**Introduction**

The Human Tissue Authority (HTA) was established in 2005. All research establishments in the United Kingdom that conduct human biobanking are required to obtain a license from the HTA to operate. Since its inception, the HTA has inspected 158 establishments working with human tissue and has made public and accessible all the reports.^1^ These reports, therefore, provide a useful source of information about biobanks in the United Kingdom.

The goal of this master's thesis concept is to analyze the type and frequency of deficiencies in biobanks seeking licensing from a national licensing body.

**Methods**

We have set out to review HTA reports to identify the most important areas where shortfalls were identified in biobanks before licensing.

**Results**

The results after analyzing 158 of these reports have shown that the research sector in the United Kingdom is mostly compliant with the standards^2^ from the authority. However, 52 major shortfalls and 329 minor shortfalls were identified in 4 distinct operational areas and these are described hereunder.

There were 13 major shortfalls reported in the consent (C) standard as the result of several deficiencies in the consent process and related documentation and the illegal storage of human tissue, e.g., ethical approval had expired or when samples were stored beyond the agreed time. They also were identified when untrained staff were seeking consent and when consent training was inadequate. Moreover, 40 minor shortfalls were reported in the C standard. They occurred due to the lack of documentation, standard operating procedures (SOPs) not reflecting current practices, establishments not offering refresher training to those seeking consent, when erroneous information was given at the training sessions, and when staff competency was not being assessed.

There were 13 major shortfalls reported in the Governance and Quality System (GQS) standard. They were related to insufficient SOPs and policies, lack of risk assessments, no provisions for back‐up of records, insufficient internal audits, and unresolved corrective action plan. Poor staff training, lack of a system to maintain records, and no restriction to access patient information, all led to major shortfalls. There were 169 minor shortfalls reported in the GQS standard.

They were reported when there were not enough risk assessments performed for all practices, when there were incomplete or inconsistent internal audit schedules, and when there was a lack of follow‐up actions after findings were reported. Not having a review mechanism for key documents, sufficient and meaningful governance meetings, adverse event reporting, and staff training records were also reported as minor shortfalls.

There were 16 major shortfalls reported in the traceability (T) standard. They were in relation to the inability to maintain a robust sample audit trail. They spanned organizations using different traceability systems ranging from paper‐based records, proprietary software databases to commercially available sample tracking laboratory information management system. In addition, the lack of unique sample identification codes, which is known to hinder sample traceability by enabling the possibility of having duplicate IDs used for the same sample, and not having documentation with regard to sample destruction were also reported as a major shortfall.

There were 67 minor shortfalls reported in the T standard. They were in relation to the sample audit trail and when samples did not match their recorded storage location or vice versa. They were also reported when either the date, reason, or method used to dispose of a sample was not recorded. Lastly, minor shortfalls were reported when sample traceability was not maintained when samples were in transit.

There were 10 major shortfalls reported in the premises, facilities, and equipment (PFE) standard. They were reported when there was a lack of temperature monitoring of the storage areas, when establishments failed to investigate temperature deviations and trends, and when there was a lack of oxygen monitoring in storage areas using liquid nitrogen, which posed a risk to staff. There were also cases where storage conditions were insufficient or unsuitable; when there was not an alarm call‐out process when there was no challenge of alarms to verify whether they were operational; when establishments had no service contracts covering maintenance, validation, and calibration of freezers in place; and when there were no equipment checks performed.

There were 53 minor shortfalls reported in the PFE standard. These were in relation to the lack of documented cleaning and decontamination procedures of the facilities storing human tissue when there were inadequate contingency plan arrangements, samples stored in nonsecure locations, and the lack of a process to report equipment problems.

**Discussion**

The authority stands for the fundamental principles of the U.K. Human Tissue Act 2004: informed consent, dignity, quality, and openness. It also gives confidence to the public and helps establishments to bring about improvement. Keeping abreast of their published reports also ensures a deeper understanding of the HTA requirements.

**References**

1. Human Tissue Authority. The regulator for human tissue and organs. Available from: https://www.hta.gov.uk/ [Last accessed: December 15, 2021].

2. Human Tissue Authority. Research Standards and Guidance. 2016. Available from: https://content.hta.gov.uk/sites/default/files/2020-1/Code%20E%20standards.pdf [Last accessed: December 15, 2021].

Address correspondence to:

*Berthold* *Huppertz, PhD*

*Head of Gottfried Schatz Research Center*

*Medical University of Graz*

*Division of Cell Biology, Histology & Embryology*

*Gottfried Schatz Research Center*

*Graz*

*Austria*

*E‐mail*: berthold.huppertz@medunigraz.at

## Supplementary Material

Supplemental data

