# Supplementary material for: Brief Reports Prepared by Students and Graduates of Master of Science in Biobanking
Source: Biopreserv Biobank. 2023 Feb 15;21(1):98–105. doi: 10.1089/bio.2022.0064 (PMC9963501; doi:10.1089/bio.2022.0064)
Supplement: Supplemental data [file Supp_Data.docx]

**Moving towards personalized medicine with oncological biobanks: University-based biobanking model of international multicenter cooperation**
Anna Michalska-Falkowska^1,2^, Karine Sargsyan^3,4,5^

^1^Department of Clinical Molecular Biology, Medical University of Bialystok, Poland

^2^Indivumed GmbH, Hamburg, Germany

^3^International Biobanking and Education, MSc in Biobanking, Medical University of Graz, Graz, Austria

^4^Department of Medical Genetics, Yerevan State Medical University, Yerevan, Armenia

^5^National Medical Research Radiological Centre of the Ministry of Health of the Russian Federation

**Corresponding author:** Prof. Dr. med. Karine Sargsyan, MD; International Biobanking and Education, MSc in Biobanking, Medical University of Graz, Graz, Austria

E-mail: [karine.sargsyan@medunigraz.at](mailto:karine.sargsyan@medunigraz.at)

**ORCID-Numbers:**

Anna Michalska-Falkowska: 0000-0002-4859-4157

Karine Sargsyan: 0000-0001-5853-4994

**Journal:** Biopreservation and Biobanking

**Short communication:** No abstract, main 750 words, 1 tables/ figures, 15 refs

Word count Main Text: (723 / 600 to max. 750 words)

Number of Tables / Figures: (1/ 1)

References: (6 / 15)

Short running title: Oncological biobanking for personalized medicine

**Keywords** (max 3-10)**:** Biobank, Human biospecimens, Personalized medicine, Multicenter cooperation, BBMRI-ERIC Directory, BBMRI.pl, Polish Biobanking Network, BioBank Management System, Poland

**MAIN TEXT**

# INTRODUCTION

Ten years ago Hewitt (1) identified biobanking as the essential component for the acceleration of personalized medicine. Nowadays, standardized collections are widely sought after in biomedical research, and this approach generates multiple challenges since studies focused on the identification of novel biomarkers and potential drug targets often require large sets of samples for proper variable selection (2). Importantly, biological material used for high-throughput assays should be collected, preserved, and stored under strictly controlled conditions to minimize the impact of pre-analytic variables to the final results of studies that are extremely sensitive to minor, subtle changes in the molecular composition of samples (3). Yet, despite this necessity, biobanks are characterized by a high degree of heterogeneity due to the variations in the available infrastructure, used procedures, methods of quality control, components of information technology, and level of education of personnel (4).

# METHODS

***2.1 Literature review of the role of biobanking in personalized medicine***

To address the most recent status of oncology-oriented biobanks and the impact of their activity on the development of personalized medicine, a comprehensive literature review was performed. This analysis focused on the significance of biobanking to provide high-quality samples for implementation of novel diagnostic and therapeutic methods and usage of biobanked samples and data in the research.

## 2.2 Analysis of biobanks resources and their participation in research projects

Extracted literature data will be augmented with a broad study of particular oncological collections in European biobanks, including three tools: internal BioBank Management System (BBMS), on-line application of Polish Biobanking Network, and BBMRI-ERIC Directory tool. The analysis will include the determination of the number and types of biospecimens among disease-specific biobanks, biobanking activities held within collection networks and biobank networks.

## 2.3 Description of multicenter cooperation model established in MUB Biobank

Based on our own experience with establishing the Oncology Section in the Biobank at Medical University of Bialystok, Poland (MUB Biobank), including the development of a Quality Management System, creation of the business model, and coordinating the cooperation between multiple academic and industry partners, a description of a multicenter cooperation model will be included (5).

# RESULTS

***3.1 Literature review of the role of biobanking in personalized medicine***

The investigation focused on review of the current literature allowed us to collect data on the current state-of-art regarding the impact of the activity of biobanks on the development of personalized medicine.

## 3.2 Analysis of biobanks resources and their participation in research projects

Analysis of biobank resources using the online tools will serve as a basis to characterize the most recent status of oncology-oriented biobanks, their participation in networks activities and scientific projects, along with available types of biospecimens and data.

## 3.3 Description of multicenter cooperation model established in MUB Biobank

The Oncology Section in the MUB Biobank was founded in 2016 and since then has served as the main facility to provide retrospective and prospective collections of omics-grade biospecimens and comprehensive, validated sets of data within Podlasie voivodeship. The Quality Management System in MUB Biobank was designed and implemented on the basis of ISO 9001:2015 norm. Standard Operating Procedures regulating the process of biobanking within the Oncology Section in MUB Biobank were primarily prepared in cooperation with an industrial partner and are reviewed on an annual basis and updated according to the international standard ISO 20387:2018, Quality Standards for Polish Biobanks (Second Edition), and ISBER Best Practices (Fourth Edition). What is crucial for the sustainability of MUB Biobank, is that its resources are requested by national and international academic institutions, as well as foreign industry partners. Currently, the MUB Biobank team has established a workflow for the collection of viable tumor cells from pleural effusion in lung cancer patients and ascites in ovarian cancer patients. This protocol will serve as a fundamental set-up for observational studies held in cooperation with a commercial partner.

# DISCUSSION

The importance of biobanking for academics and commercial sectors is beyond question since the modern research techniques rely on high-quality biospecimens and broad sets of data. Human samples and data collected under standardized conditions are fundamental for running assays and studies focused on the identification of disease biomarkers, molecular characterization of multiple types of malignant tumors, investigations of promising drug molecules for implementation of targeted therapies.

Cooperation between biobanks and biomedical companies usually is based on situations when the biobank serves as a resource of defined biospecimens and data, and projects in which the biobank is competent in the field of sample logistics, a common practice in the clinical trials (6).

Glossary and Abbreviations

BBMRI-ERIC Biobanking and Biomolecular Resources Research Infrastructure – European Research Infrastructure Consortium

BBMRI.pl Polish National Node of BBMRI-ERIC

BBMS BioBank Management System

ISBER International Society for Biological and Environmental Repositories

MUB Biobank Biobank at Medical University of Bialystok, Poland

List of figures

Figure 1. Integration of MUB Biobank in the multicenter research workflow.


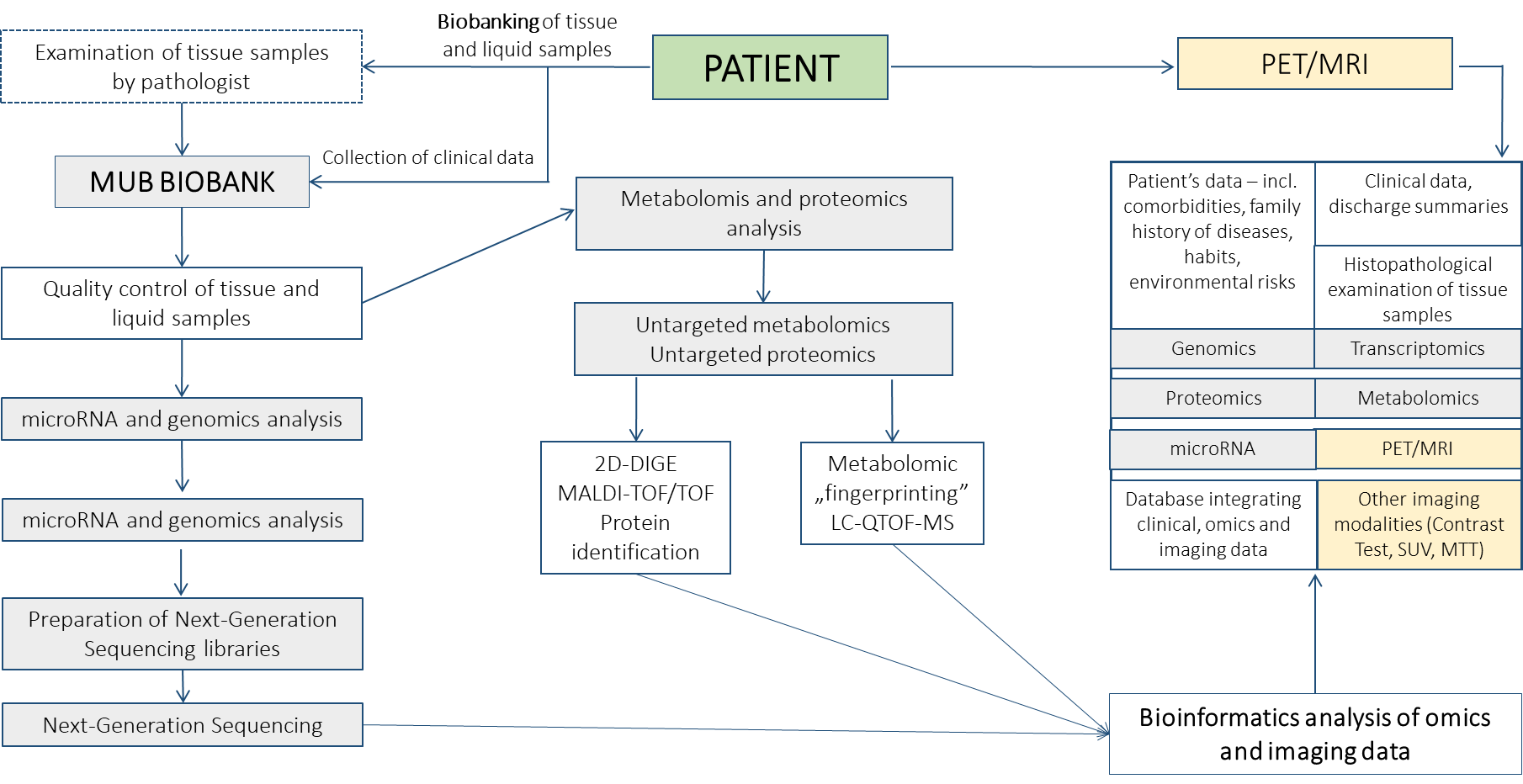


Competing interests

The authors declare that they have no competing interests.

Funding

This study was supported by the National Centre for Research and Development in the framework of the program ‘Prevention practices and treatment of civilization diseases’ – STRATEGMED (contract no. STRATEGMED2/266484/2/NCBR/2015).

The funding bodies have no role in the design of the study and collection, analysis, and interpretation of data and in writing this paper.

Ethics approval and consent to participate

The Medical University of Bialystok Commission for Research Ethics has approved the research methodology and Informed Consent Forms (approval R-I-002/357/2014).

Authors' contributions

AMF: Conceptualization, Design, Methodology, Formal analysis, Data acquisition, analysis and interpretation, Project management, Writing the paper in all stages, Visualization.

KS: Supervision, Conceptualization, Design, Methodology, Reviewing and Editing.

All authors have approved the submitted version and have agreed both to be personally accountable for the author's own contributions and to ensure that questions related to the accuracy or integrity of any part of the work, even ones in which the author was not personally involved, are appropriately investigated, resolved, and the resolution documented in the literature.

# Reference list

1. Hewitt RE. Biobanking: the foundation of personalized medicine. Curr Opin Oncol. 2011 Jan;23(1):112-9. doi: 10.1097/CCO.0b013e32834161b8.
2. Lommen K, Odeh S, Theije CC, Smits KM. Biobanking in Molecular Biomarker Research for the Early Detection of Cancer. Cancers (Basel). 2020 Mar 25;12(4):776. doi: 10.3390/cancers12040776.
3. Zhou JH, Sahin AA, Myers JN. Biobanking in genomic medicine. Arch Pathol Lab Med. 2015 Jun;139(6):812-8. doi: 10.5858/arpa.2014-0261-RA.
4. Ransohoff DF, Gourlay ML. Sources of bias in specimens for research about molecular markers for cancer. J Clin Oncol. 2010 Feb 1;28(4):698-704. doi: 10.1200/JCO.2009.25.6065.
5. Niklinski J, Kretowski A, Moniuszko M, Reszec J, Michalska-Falkowska A, Niemira M, Ciborowski M, Charkiewicz R, Jurgilewicz D, Kozlowski M, Ramlau R, Piwkowski C, Kwasniewski M, Kaczmarek M, Ciereszko A, Wasniewski T, Mroz R, Naumnik W, Sierko E, Paczkowska M, Kisluk J, Sulewska A, Cybulski A, Mariak Z, Kedra B, Szamatowicz J, Kurzawa P, Minarowski L, Charkiewicz AE, Mroczko B, Malyszko J, Manegold C, Pilz L, Allgayer H, Abba ML, Juhl H, Koch F; MOBIT Study Group. Systematic biobanking, novel imaging techniques, and advanced molecular analysis for precise tumor diagnosis and therapy: The Polish MOBIT project. Adv Med Sci. 2017 Sep;62(2):405-413. doi: 10.1016/j.advms.2017.05.002.
6. Baber R, Hummel M, Jahns R, von Jagwitz-Biegnitz M, Kirsten R, Klingler C, Nussbeck SY, Specht C. Position Statement from the German Biobank Alliance on the Cooperation Between Academic Biobanks and Industry Partners. Biopreserv Biobank. 2019 Aug;17(4):372-374. doi: 10.1089/bio.2019.0042. Epub 2019 Jul 17. PMID: 31314575; PMCID: PMC6703240

**Standardization in Biobanking: Guide to implement ISO 20387:2018 in biobanks certificated with ISO 9001:2015**

Michael Zúñiga^1^, Karine Sargsyan^2,3,4^

^1^Agencia Costarricense de Investigaciones Biomédicas, Biobank, San José, Costa Rica

^2^International Biobanking and Education, MSc in Biobanking, Medical University of Graz, Graz, Austria

^3^Department of Medical Genetics, Yerevan State Medical University, Yerevan, Armenia

^4^National Medical Research Radiological Centre of the Ministry of Health of the Russian Federation

**Corresponding author:** Prof. Dr. med. Karine Sargsyan, MD; International Biobanking and Education, MSc in Biobanking, Medical University of Graz, Graz, Austria

E-mail: [karine.sargsyan@medunigraz.at](mailto:karine.sargsyan@medunigraz.at)

**Journal:** Biopreservation and Biobanking

**Short communication:** No abstract, main 750 words, 1 tables/ figures, 15 refs

Word count Main Text: (361 to max. 750 words)

Number of Tables and Figures: N/A

References: (9 / 15)

Short running title: ISO20387 Implementation guide

**Key words** (max 3-10)**:** Biobank standardization, ISO 20387:2018, Biobanking

**MAIN TEXT**

# INTRODUCTION

Current biomedical research is based on defined study groups with high quality samples and data, collected from various biobanks and bio-clinical studies (1). For this reason, the collection, processing, storage and management of biological data and samples in biobanks must be harmonized.

Standardization can contribute significantly to solving this harmonization with the aim of disseminating the results of research and innovations, as well as publications and patents (2).

Biobanks have been seen to be in a debate on the implementation of ISO 9001 or the ISO1700 series to standardize all their processes. Due to the nature of each standard (QMS - ISO 9001 and the assessment of competence - ISO 17000), experience indicates the need for a combined and expanded approach to be able to include specific biobank requirements as inISO 20387, the first ISO standard specific to Biobanks. This standard focuses not only the operational process of the biobank involved, but also evaluates the competence to carry out the specific task of the biobank (3,4,5,6).

Currently most of the biobanks maintain ISO 9001: 2015 or 17025: 2017 certification, even though it is 3 years since the publication of the specific standard for Biobanks (ISO 20387: 2018). Of the 622 biobanks that constitute the BBMRI-ERIC, only 24 biobanks have any certification: 2 with ISO 15189 certification, 2 with ISO 17025 & ISO 15189, 18 with ISO 9001, 1 with ISO 9001 & ISO 17025 and only one with ISO 20387(7,8,9).

The goal of this Master’s Thesis concept is to develop a guide to assist biobanks to progress from ISO 9001:2015 to the ISO 20387:2018 standard.

# METHODS

The method to be used will be a review of publications on the implementation of both standards in biobanks, in order to make an objective comparison of all the processes involved and be able to create a guide that can be implemented in biobanks certified with ISO 9001: 2015 to obtain ISO 20387 certification.

# RESULTS

During the development of this thesis, it has been possible to show that if a biobank certified with ISO 9001 in all its processes of collection, acquisition, preparation, analysis, storage and distribution of biological material and its associated information, as well as competent personnel, the implementation of the ISO 20387 standard could be carried out in the short term.

**Glossary and Abbreviations**

ISO International Organization for Standardization

QMS Quality Management System

Competing interests

The authors declare that they have no competing interests

# REFERENCE LIST

1. Annaratone L, De Palma G, Bonizzi G, Sapino A, Botti G, Berrino E, Mannelli C, Arcella P, Di Martino S, Steffan A, Daidone MG, Canzonieri V, Parodi B, Paradiso AV, Barberis M, Marchiò C; Alleanza Contro il Cancro (ACC) Pathology and Biobanking Working Group. (2021): Basic principles of biobanking: from biological samples to precision medicine for patients. Virchows Arch, 479(2):233-246. doi: 10.1007/s00428-021-03151-0
2. Meinung, B; Martin, D. & Zimmermann, U. (2019): Standardization in biobanking – between cooperation and competition, J Lab Med, 43(6): 317–328
3. Bosscha, M (2017): The future ISO standard for management systems and quality of biobanks [Power Point Presentation]. Embric https://www.embric.ugent.be/sites/default/files/5MarleenBosschaertsNew%20biobank%20standard.pdf
4. Davis E, Hampson K, Bray C, Dixon K, Ollier B, and Yuille M. (2012): Selection and Implementation of the ISO9001 Standard to Support Biobanking Research Infrastructure Development. Biopreservation and Biobanking. 10(2):162-7.doi: 10.1089/bio.2011.0044
5. De Blasio, P & Biunno, I. (2021): New Challenges for Biobanks: Accreditation to the New ISO 20387:2018 Standard Specific for Biobanks. BioTech, 10, 13. https://doi.org/10.3390/biotech10030013
6. Mouttham L, Garrison S, Archer D & Castelhano M. (2021) [A Biobank's Journey: Implementation of a Quality Management System and Accreditation to ISO 20387](https://www.liebertpub.com/doi/full/10.1089/bio.2020.0068). Biopreservation and Biobanking, 19 (3): 163-170 <https://doi.org/10.1089/bio.2020.0068>
7. BBMRI-ERIC (2021): BBMRI-ERIC Directory [Database]. Retrived from <https://directory.bbmri-eric.eu/menu/main/background>
8. Ferdyn, K *et al.*(2019): Quality Management System in the BBMRI.pl Consortium: Status Before the Formation of the Polish Biobanking Network. Biopreservation and Biobanking, 17 (5): 401-409
9. Irish National Accreditation Board (2021): Accreditation in Biobanks according to ISO20387. [Power Point Presentation]. INAB Biobanking Webinar <https://www.youtube.com/watch?v=qMEBf_h9Zk8>

**Benchmarking study of animal biobanks in Europe**

Mbayame Diop^1^, Paolo Bonvicini^1^, Samantha Luciano^1^, Tommy-Lee Banlier^1^, Youmna Chelbi^1^, Paul Hofman^1,2^, Nicole Arrighi^1,3^

^1^MSc Biobanks and Complex Data Management, University Côte d’Azur, Nice, France

^2^Biobank 0033-00025 and FHU OncoAge, Nice Hospital Center, University Côte d’Azur, Nice, France

^2^INSERM U1065, Centre Méditerranéen de Médecine Moléculaire, Université Côte d’Azur, Nice, France

**Corresponding author**: Dr Nicole Arrighi, Lecturer, MSc Biobanks and Complex Data Management, University Côte d’Azur, Nice, France

E-mail: nicole.arrighi@univ-cotedazur.fr

**Journal:** Biopreservation and Biobanking

**Short communication:** No abstract, main 750 words, 1 table/ figure, 15 refs

Word count Main Text: (663 to max. 750 words)

Number of Tables and Figures: (1/ 1)

References: (8 / 15)

Short running title: Animal biobanking

**Key words** (max 3-10): Animal biobanks, European biobanks, biobank quality management, biobank regulatory compliance, biobank research Impact

**MAIN TEXT**

# INTRODUCTION

Animal biobanks remain relatively obscure to the public but play a crucial role in research and focus on all animal species. Despite being present in various sectors of activity (i.e., therapeutics, breeding, industry, ecology, biodiversity, biomedical research, etc.), this domain is poorly represented in publications [1]. The aim of this paper was to run a benchmarking analysis to target European animal biobanks.

Overall, benchmarking can be defined as the process of measuring key metrics and practices and comparing them to understand how an organization should improve its performance [2]. It is classified according to selected metrics and types of comparisons as:

- internal (applied to different departments within a company);

- external (compared to a competitor);

- functional (conducted between companies from different markets);

- horizontal (compared similar processes in any sector of activities).

Specifically, this study applied horizontal benchmarking through four prominent European animal biobanks.

# METHODS

## Biobanking indicators

To apply benchmarking to different public and private European animal biobanks, web research was framed to establish a set of accessible indicators to rely on and therefore identify the biobanks of interest: quality management, regulatory compliance, research impact, and fundings. The following step consisted of creating two groups of comparisons, a public-public and a public-private, based on vision, mission, and accessibility of previously chosen indicators.

## European veterinary biobanks selection

Thanks to the research methodology, we have identified and chosen three public biobanks: *Zoologisches Forschungsmuseum A. Koenig* (ZFMK, Bonn, Germany), the *European Association of Zoos and Aquaria* (EAZA, Amsterdam, the Netherlands), the *Biomedical Primate Research Centre* (BPRC, Rijswijk, the Netherlands), and a private one, the *VetBioBank* (VBB, Marcy-l'Étoile, France):

- ZFMK, is part of the Leibniz Institute for analysis of biodiversity (LIB). It counts more than 5 million frozen samples in zoological collections, used for non-commercial molecular biodiversity research [3].
- EAZA is a recognized organization for animal welfare research and application [4].
- BPRC is a not-for-profit scientific research institute aiming to deepen knowledge in human diseases. It is part of the **European infrastructure for translational medicine (EATRIS,** Amsterdam, The Netherlands**)** [5].
- VBB l focuses on veterinary regenerative medicine using neonatal stem cells against inflammatory diseases [6].

For all these biobanks, we established a set of accessible indicators: quality management, regulatory compliance, research impact, and funding.

# RESULTS

## Biobank quality management

Horizontal benchmarking portrays overall adherence to harmonization, standardization processes, and standard operating procedures. Concerning regulatory compliance, public biobanks are subject to supranational regulations such the as Nagoya Protocol, CITES, European directive 2010/63, and Material transfer agreements (MTAs) while the private biobank only refers to ethics of stem cells usage (Table I).

## Research impact and international visibility

The impact factor is related to international networking (GGBN, Frozen Ark, EAZA, etc.) and international peer-reviewed publications referenced in the NCBI database; VBB clearly highlights the number of publications and collaborations. Lastly, in terms of finance, all the biobanks under analysis are mainly supported by European permanent and punctual funders, private donors and foundations.

# DISCUSSION

This study confirms the similarities between veterinary and human biobanking objectives in terms of quality management and human research acceleration [7, 8]. Moreover, it highlighted to what extent these four biobanks differ, considering four metrics: quality management, regulatory compliance, research impact, and finance (Table I).

Public biobanks seem to rely on well-defined quality management frameworks characterized by clear standardization and harmonization practices and strong adherence to regulatory compliance. The involvement in international networks is usually linked with higher scientific impact factors, a trend to be found in finance also: European, national, and permanent/punctual funders increase the chances of receiving grants.

About the private biobank, no high authority or reference was found regarding quality management and regulatory compliance and laws and standards on which it is based are unknown.

To summarize, accessibility of indicators should be improved through communication channels and further studies focusing on the comparison between public and private animal biobanking are crucial to increase the degree of awareness among the general population, scientists, and future providers.

Glossary and Abbreviations

BPRC Biomedical Primate Research Centre

CBD Convention on Biological Diversity

CITES Commerce international des espèces sauvages

**EATRIS** **European infrastructure for translational medicine**

EAZA European Association of Zoos and Aquaria

GGBN Global Genome Biodiversity Network

LIB Leibniz institute for analysis of biodiversity

MTA Material Transfer Agreement

NCBI National Center for Biotechnology Information

SOPs Standard operation procedures

VBB VetBioBank

ZFMK Zoologisches Forschungsmuseum A. Koenig

ZIMS Zoo aquarium animal management software

List of tables

| **Biobanks** | ZFMK/LIB | EAZA | BPRC | VBB |
| --- | --- | --- | --- | --- |
| **Quality management** | Synthesis+ project for harmonization and standardization  Internal SOPs | ZIMS database  Internal SOPs  EAZA accreditation program | High quality samples and protocols | Good manufacturing practices  Quality manager and technician |
| **Regulatory compliance** | International (Nagoya, CITES, CBD)  European  National  Local | International  (Nagoya, CITES) | International (CITES)  European (Directive 2010/63/EU)  MTA | Ethics (neonatal stem cells) |
| **Research impact**  *NCBI database* | International network (GGBN, DNA Bank network, Frozen Ark)  206 scientific publications since 2002 | International network (EAZA)  Research on worldwide protection  19 scientific publications since 2012 | Willing of reducing animal tests  Improve medical research | Collaboration in many fields  5 clinical studies  1600 treatments provided  17 scientific publications since 2014 |
| **Fundings** | Permanent  Punctual  Donations | Permanent  Punctual | European  National  Private fundations  Donations | European  National  Local funders |

**Table 1: Comparison of different animal biobanks** on quality management, regulatory compliance, research impact and fundings. There are 3 public biobanks: the *Zoologisches Forschungsmuseum A. Koenig* (ZFMK), the *European Association of Zoos and Aquaria* (EAZA), the *Biomedical Primate Research Centre* (BPRC), and a private one, the *VetBioBank* (VBB).

**Acknowledgements**

We thank the team of the Master of Science in Biobanking and Complex Data Management, the University of Cote D’Azur, and the lectures who all contributed to our enrichment as future graduates and the given opportunity to further research through personal contributions and points of view.

**Competing interests**

The authors declare that they have no competing interests

**Authors' contributions**

All authors equally contributed to Conceptualization, Design, Methodology, Formal analysis, Data curation, Project administration, Writing - all stages, Visualization.

All authors have approved the submitted version and have agreed both to be personally accountable for the author's own contributions and to ensure that questions related to the accuracy or integrity of any part of the work, even ones in which the author was not personally involved, are appropriately investigated, resolved, and the resolution documented in the literature.

# Reference list

1. Groeneveld LF, Gregusson S, Guldbrandtsen B, Hiemstra SJ, Hveem K, Kantanen J, Lohi H, Stroemstedt L, Berg P. Domesticated Animal Biobanking: Land of Opportunity. *PLoS Biol*. 2016 Jul 28;14(7):e1002523. doi: 10.1371/journal.pbio.1002523. PMID: 27467395; PMCID: PMC4965055.
2. Vaught J, Kelly A, Hewitt R. A review of international biobanks and networks: success factors and key benchmarks. *Biopreserv Biobank*. 2009 Sep;7(3):143-50. doi: 10.1089/bio.2010.0003. Epub 2010 Mar 17. PMID: 24835880; PMCID: PMC4046743.
3. Williams N. Frozen ark to hold samples of endangered species. *Curr Biol*. 2004 Aug 24;14(16):R638-9. doi: 10.1016/j.cub.2004.08.003. PMID: 15324674
4. Moresco A, Feltrer-Rambaud Y, Wolfman D, Agnew DW. Reproductive one health in primates. *Am J Primatol*. 2021 Sep 13:e23325. doi: 10.1002/ajp.23325. Epub ahead of print. PMID: 34516669.
5. de Groot NG, Heijmans CMC, Bontrop RE. AIDS in chimpanzees: the role of MHC genes. *Immunogenetics.* 2017 Aug;69(8-9):499-509. doi: 10.1007/s00251-017-1006-6. Epub 2017 Jul 10. PMID: 28695283
6. Rakic R, Bourdon B, Demoor M, Maddens S, Saulnier N, Galéra P. Publisher Correction: Differences in the intrinsic chondrogenic potential of equine umbilical cord matrix and cord blood mesenchymal stromal/stem cells for cartilage regeneration. *Sci Rep*. 2020 Jul 21;10(1):12075. doi: 10.1038/s41598-020-69170-0. Erratum for: Sci Rep. 2018 Sep 14;8(1):13799. PMID: 32694679; PMCID: PMC7374704.
7. Hostetter G, Collins E, Varlan P, Edewaard E, Harbach PR, Hudson EA, Feenstra KJ, Turner LM, Berghuis BD, Resau JH, Jewell SD. Veterinary and human biobanking practices: enhancing molecular sample integrity. Vet Pathol. 2014 Jan;51(1):270-80. doi: 10.1177/0300985813510532. Epub 2013 Nov 13. PMID: 24227009.
8. Castelhano MG, Creevy KE, Mullins PF. How veterinary biobanking provides opportunities to accelerate research. *J Am Vet Med Assoc*. 2018 Nov 15;253(10):1243-1244. doi: 10.2460/javma.253.10.1243. PMID: 30398415.

**SITOGRAPHY**

**LIB** - <https://bonn.leibniz-lib.de/en>

**EAZA** - <https://www.eaza.net/conservation/research/eaza-biobank/>

**BPRC** - <https://www.bprc.nl/en/biobank>

**VBB** - [https://www.vetbiobank.com](https://www.vetbiobank.com/)

**Environmental Sustainability in Biobanking**

Anita Litschauer^1^, Berthold Huppertz^2^

^1^HVD Life Sciences Vertriebs GmbH, Vienna, Austria

^2^Head of Gottfried Schatz Research Center, Head of Cell Biology, Histology and Embryology, Head of University Training Course MSc in Biobanking, Medical University of Graz, Graz, Austria

**Corresponding author:** Univ.-Prof. Dr.rer.nat. Berthold Huppertz; Cell Biology, Histology and Embryology, Medical University of Graz, Graz, Austria

E-Mail: [berthold.huppertz@medunigraz.at](mailto:berthold.huppertz@medunigraz.at)

**ORCID-Numbers:**

Berthold Huppertz: 0000-003-4814-2158

**Journal:** Biopreservation and Biobanking

**Short communication:** No abstract, main 750 words, 1 tables/ figures, 15 refs

Word count Main Text: (447 / 600 to max. 750 words)

Number of Tables and Figures: (0/ 1)

References: (6 / 15)

Short running title: Environmental Sustainability in Biobanking

**Key words** (max 3-10): Biobank Storage, Ultra-Low Temperature Sample Storage, Efficiency of Freezers in Biobank, human biobank

Main Text

1. Introduction

**Opportunities to reduce the environmental impact of manual Ultra-Low Temperature (ULT) sample storage**

Sustainability is omnipresent and confronts us today on many levels of our lives. A number of environmental initiatives have been started to contribute to the reduction of the environmental footprint in the healthcare sector, including initiatives in the field of laboratory practices (1). Biobanks with their main activities including the collection, preservation, processing, storage, and distribution of samples and related data have recently attracted attention in the field of laboratory sustainability (2). Robust storage conditions are crucial to ensure high-quality and ‘fit for purpose’ samples in biobanking and therefore ultra-low storage temperatures, achieved in so-called ultra-low temperature (ULT) freezers, are common for long-term storage in biobanking (3). ULT freezers are known to be one of the most energy-intensive types of equipment in laboratories (4), while biobanks storing large sample collections may operate hundreds of ULT freezers in their facilities.

1. Methods

With the overall aim of encouraging biobanks to consider practices contributing to the Sustainable Development Goals (SDGs) and to emphasize the topic ‘Environmental Sustainability in Biobanking’, 24 university-based sustainability programs (Green Lab Programs) were selected for evaluation to identify actions to meet environmental responsibility related to sample storage and ULT freezers. 31 actions in the form of measures, recommendations, and considerations towards more sustainable sample storage at ultra-low temperatures were identified within the topics of sample management, freezer management, freezer maintenance, and procurement considerations.

1. Results

The most frequently found actions include actions related to reducing the energy consumption of ULT freezers including purchasing energy-efficient ULT freezers that meet certain criteria, like holding an Energy Star label (5), and the ‘Chill-Up’ of ULT freezers by increasing the set point temperature from -80°C to -70°C. In addition to the approach of reducing the energy consumption of ULT freezers in use, several actions promoted have in common the aim to reduce the number of ULT freezers needed, including room temperature sample storage (RTSS) and ‘Clear-Out’ of unused and unnecessary samples.

The actions identified were compared to the latest available ISBER Best Practices (6) and showed that the related subject areas of two-thirds of actions found in the university-based sustainability programs were mentioned in ISBER Best Practices, although partly in a different context.

1. Discussion

The activities of biobanks are crucial for the progress in biomedical research, ultimately contribute to better health care, and therefore play an important and responsible role for our society. The implementation of environmental sustainability in their operations would further contribute to the goals of sustainable development. On the example of ultra-low temperature sample storage, it is shown that there is room for improvement through the implementation of a number of sustainable practices and considerations.

1. Reference List
2. Budd K. *Hospitals race to save patients — and the planet*. Association of American Medical Colleges AAMC. Available from: <https://www.aamc.org/news-insights/hospitals-race-save-patients-and-planet> [Accessed: 6th November 2019].
3. University College London. *Sustainable biobanking*. Available from: <https://www.ucl.ac.uk/greenucl/case-studies/2019/mar/sustainable-biobanking> [Accessed: 20th November 2019].
4. Mendy M, Caboux E, Lawlor RT, Wright J, Wild CP. *IARC Technical Publication No.44, Common minimum technical standards and protocols for biobanks dedicated to cancer research*. International Agency for Research on Cancer (IARC), 2017. Available from: <https://publications.iarc.fr/Book-And-Report-Series/Iarc-Technical-Publications/Common-Minimum-Technical-Standards-And-Protocols-For-Biobanks-Dedicated-To-Cancer-Research-2017> [Accessed: 16th January 2020].
5. Paradise A. *Market Assessment of Energy Efficiency Opportunities in Laboratories*. Center for Energy Efficient Laboratories (CEEL), 2015. Available from: <https://www.etcc-ca.com/sites/default/files/reports/ceel_market_assessment_et14pge7591.pdf> [Accessed: 22nd September 2019].
6. Environmental Protection Agency, Department of Energy. *Laboratory Grade Refrigerators and Freezers Specification Version 1.0 ENERGY STAR.* Available from: <https://www.energystar.gov/products/spec/laboratory_grade_refrigerators_and_freezers_specification_version_1_0_pd> [Accessed: 17th April 2020].
7. International Society for Biological and Environmental Repositories (ISBER). *ISBER Best Practices: Recommendations for Repositories Fourth Edition*. ISBER, 2018. Available from: <https://www.isber.org/page/BPR> [Accessed: 16th January 2020].

**Raising the awareness of biobanking in the Swiss population through higher education**

Nesa Marti^1^, Karine Sargsyan^2,3,4^

^1^Translational Research Coordination, European Thoracic Oncology Platform (ETOP), Bern, Switzerland.

^2^International Biobanking and Education, MSc in Biobanking, Medical University of Graz, Graz, Austria

^3^Department of Medical Genetics, Yerevan State Medical University, Yerevan, Armenia.

^4^National Medical Research Radiological Centre of the Ministry of Health of the Russian Federation.

**Corresponding author:** Prof. Dr. med. Karine Sargsyan, MD; International Biobanking and Education, MSc in Biobanking, Medical University of Graz, Graz, Austria

E-mail : [karine.sargsyan@medunigraz.at](mailto:karine.sargsyan@medunigraz.at)

**Journal:** Biopreservation and Biobanking

**Short communication:** No abstract, main 750 words, 1 tables/ figures, 15 refs

Word count Main Text: (365)

Number of Tables and Figures: (0/ 1)

References: (1/ 15)

Short running title: Swiss Education in Biobanking

**Key words** (max 3-10)**:** Biobanking, Education, Public Health

**MAIN TEXT**

1. INTRODUCTION

The change from medical treatments for the “average patient” towards individualized therapy is the focus of today's medicine. In recent years, the promotion of personalized medicine has also become an increasingly important topic in Switzerland, leading to the establishment of the Swiss Personalized Health Network (SPHN) and Swiss Biobanking Platform (SBP). Despite the existence of this governmental organization, the path to individual treatment can only be achieved together with the Swiss population; Medical research depends on the willingness of many to share their health data and genetic information and to donate their biological samples. Brall et al., (2021) reported in their survey on “Swiss public’s willingness to participate in personalized health research and biobanking”, that research on personalized health is supported by only slightly more than half of the Swiss population. The restraint of many survey participants could be traced back to concerns about discrimination, confidentiality, misuse of data and samples – and to the lack of knowledge about biobanking.

1. METHODS

One approach to expand this knowledge, and thus increase the willingness to donate biospecimen and data to biobanks, might be by embedding the subject “Biobanking” in the curriculum of Swiss high schools. The development of a biobanking teaching unit and its implementation aims to raise awareness of biobanks among the next generation - to promote a sustainable engagement.

In collaboration with the *Bern University of Teacher Education, Bern, Switzerland* and the *Gymnasium Thun, Thun, Switzerland* an interdisciplinary teaching unit will be developed and taught.

1. RESULTS

The unit elaborates the term “Biobanking”, give an insight in personalized medicine with its basic underlying scientific methods, and discusses ethical considerations towards data protection, sample ownership and genetic testing.

This pilot project aims to provide a qualitative analysis based on tailored questionnaires to students whether, and to what extent, the teaching unit has changed the students' perception towards biobanking.

1. DISCUSSION

As the public participation in biobanking is key for the long-tern success of a biobank, we need to find ways to make public engagement sustainable. By embedding this subject in the Swiss high school curriculum and thus involving the next generation, this pilot project elaborates a possible solution to anchor the cognition of biobanking in our society.

1. REFERENCES

Brall C, Berlin C, Zwahlen M, Ormond K E, Egger M, Vayena E. (2021) Public willingness to participate in personalized health research and biobanking: A large-scale Swiss survey. PLoSONE.16(4):e0249141.

**Implementation of electronic informed consent for cancer research**

Nina Bertheussen Krüger^1,^ Karine Sargsyan^2,3,4^

^1^Divison of Cancer Medicine, Oslo University Hospital, Norway

^2^International Biobanking and Education, MSc in Biobanking, Medical University of Graz, Graz, Austria

^3^Department of Medical Genetics, Yerevan State Medical University, Yerevan, Armenia

^4^National Medical Research Radiological Centre of the Ministry of Health of the Russian Federation

**Corresponding author:** Prof. Karine Sargsyan, MD; International Biobanking and Education, MSc in Biobanking, Medical University of Graz, Graz, Austria

E-mail : [karine.sargsyan@medunigraz.at](mailto:karine.sargsyan@medunigraz.at)

**ORCID-Numbers:**

Nina Bertheussen Krüger: 0000-0003-3505-1759

Karine Sargsyan: 0000-0001-5853-4994

**Journal:** Biopreservation and Biobanking

**Short communication:** No abstract, main 750 words, 1 tables/ figures, 15 refs

Word count Main Text: (420 / 600 to max. 750 words)

Number of Tables and Figures: (0/ 1)

**References: (32 / 15)**

Short running title: Implementation of electronic informed consent for cancer research

**Key words** (max 3-10)**:** Cancer Research, Biobanking Systems, Cancer Research with Biobanking

## Main Text

1. Introduction

### Benefits and experiences

Cancer research, in general, is largely dependent on access to biobank samples, and access to large-scale biobank collections is of crucial importance for the development and implementation of precision cancer medicine. Informed consent is the fundament of all research activities, including the collection of biological samples for cancer research. It is therefore of crucial importance that there is a system in place so that each patient that is diagnosed with cancer is given the opportunity to participate in cancer research, and that the informed consent is retrieved in a systematic and trustworthy manner. Cancer patients represent a large and heterogeneous group, differing in sex, age, and physical condition, and cancer research is equally dependent on all of them to participate.

1. Methods

Although we are using electronic solutions in many aspects of our daily life, like logging on to services and communicating with our surroundings, using such systems to involve people in medical research is still a rather unexplored field. To achieve the beneficial effects, the efforts needed for the development and implementation of electronic informed consent must be mapped. The transformation to an electronic system may have unexpected consequences. This is due to the innovative nature of the concept.

1. Results

The total impact is therefore dependent on stakeholders’ perspectives and needs, design, and implementation. Cancer patients as research participants are most likely in a vulnerable situation, and we should keep in mind that the way they are asked to participate might have an impact on their willingness to contribute to cancer research. Attention to both electronic solutions and awareness when replacing the important discussion between the donor and the health care personnel with a digital solution is necessary. It has been found that there are several issues related to the design and implementation of an electronic informed consent solution. These issues run in the ethical, technical, and security-related dimensions.

Comprehensive work with design, implementation, and hospital logistics are demanded for a successful implementation, and thereby the realization of the achievable benefits of an electronic informed consent solution. It is not doubtable that society is moving into a more digitalized future, and we are moving rapidly.

1. Discussion

The use of paper forms for informed consent for cancer research should already be considered not trustworthy. Modern technology offers the opportunity of designing and implementing a further developed informed consent solution that contributes to a more streamlined process and thereby a more efficient research process, thus having the potential to be a game-changer for cancer research.

1. Reference List

1. Vollmann J, Winau R. Informed consent in human experimentation before the Nuremberg code. BMJ. 1996;313(7070):1445-1449. doi:10.1136/bmj.313.7070.1445

2. Sandu, A. and Frunza, A. Ethics in Research Practice and Innovation. Romania: IGI Global, Disseminator of Knowledge; 2009, p.171

3. Kaye, J. et.al. (2014). Dynamic consent: a patient interface for twenty-first century research networks. European Journal of Human Genetics, p.141. Available from: https://www.nature.com/articles/ejhg201471 (Assessed 3th February 2020)

4. US Food and Drug Administration. Use of Electronic Informed Consent, Questions and Answers, Guidance for Institutional Review Boards, Investigators and Sponsors, 2016

5. Regulation (EU) No 910/2014 of the European Parliament and of the Council of 23 July 2014 on electronic identification and trust services for electronic transactions in the internal market and repealing Directive 1999/93/EC. Available from: https://eur-lex.europa.eu/eli/reg/2014/910/oj (Assessed 11th June 2020).

6. The Norwegian Ministry of Local Government and Modernisation. Én digital offentlig sektor, Digitaliseringsstrategi for offentlig sektor 2019–2025, 2019 June 11. Available from: https://www.regjeringen.no/no/tema/statlig-forvaltning/ikt-politikk/digitaliseringsstrategi-for-offentlig-sektor/id2612415/ (Accessed 12th February 2020)

7. European Commission. Digital Europe Programme: A proposed €9.2 Billion of funding for 2021-2027. Last update: 26 June 2019. Available from https://ec.europa.eu/digital-single-market/en/news/digital-europe-programme-proposed-eu92-billion-funding-2021-2027 (Accessed 2nd February 2020)

8. Organization for Economic Cooperation and Development (OECD). Health in the 21st Century: Putting Data to Work for Stronger Health Systems. OECD Health Policy Studies. OECD Publishing. Paris, 2019

9. TransCelerate BioPharma. eConsent: Implementation guidance version 1 (2017). Available from: https://transceleratebiopharmainc.com/?s=eConsent (Accessed 20th February 2020)

10. Rahm AK, Wrenn M, Carroll NM, Feigelson HS. Biobanking for research: a survey of patient population attitudes and understanding. J Community Genet. 2013 Oct;4(4):445-50

11. Buckley MT, Lengfellner JM, Koch MJ, Pacheco HO, Hoidra C, Damron DJ, et al. The Memorial Sloan Kettering (MSK) electronic informed consent (eIC) platform for clinical trials: An operational model and suite of tools for obtaining informed consent, and managing consent documents. JCO. 2018 May 20;36(15_suppl):e18577

12. Chalil Madathil K, Koikkara R, Obeid J, Greenstein JS, Sanderson IC, Fryar K, et al. An investigation of the efficacy of electronic consenting interfaces of research permissions management system in a hospital setting. Int J Med Inform. 2013 Sep;82(9):854-63

13. Doerr, Megan and Suver, Christine and Wilbanks, John. Developing a Transparent, Participant-Navigated Electronic Informed Consent for Mobile-Mediated Research. April 22, 2016. Available at SSRN: https://ssrn.com/abstract=2769129 (Accessed 20th February 2020)

14. Coiera E. e-Consent: The Design and Implementation of Consumer Consent Mechanisms in an Electronic Environment. Journal of the American Medical Informatics Association. 2003 Nov 21;11(2):129-40

15. Vanaken H, Masand SN. Awareness and Collaboration Across Stakeholder Groups Important for eConsent Achieving Value-Driven Adoption. Ther Innov Regul Sci. 2019 11;53(6):724-35

16. Friedman, Batya, Edward Felten, and Lynette I. Millett. Informed consent online: A conceptual model and design principles. University of Washington Computer Science & Engineering Technical Report 00–12–2 8 (2000) Available from: https://www.vsdesign.org/publications.php (Accessed 22th February 2020)

17. Shenoy P. Electronic informed consenting: A boon to modernize consenting process. Perspect Clin Res. 2015;6(4):173

18. W. Rowan, Y. O'Connor, L. Lynch, & C. Heavin. (2017). Exploring the Situational Approach to Decision Making: User eConsent on a Health Social Network (Version 10008118), 2017

19. Balestra M, Shaer O, Okerlund J, Westendorf L, Ball M, Nov O. Social Annotation Valence: The Impact on Online Informed Consent Beliefs and Behavior. J Med Internet Res. 2016 07 20;18(7):e197

20. Clarke, Roger. eConsent: A Critical Element of Trust in eBusiness. BLED 2002 Proceedings. 12. Available from: http://aisel.aisnet.org/bled2002/12 (Accessed 20th February 2020)

21. Rothwell E, Wong B, Rose NC, Anderson R, Fedor B, Stark LA, et al. A randomized controlled trial of an electronic informed consent process. J Empir Res Hum Res Ethics. 2014 Dec;9(5):1-7

22. Frelich, Matthew J., Matthew E. Bosler, and Jon C. Gould. "Research Electronic Data Capture (REDCap) electronic Informed Consent Form (eICF) is compliant and feasible in a clinical research setting." Int J Clin Trials 2.3 (2015): 51

23. Boutin NT, Mathieu K, Hoffnagle AG, Allen NL, Castro VM, Morash M, et al. Implementation of Electronic Consent at a Biobank: An Opportunity for Precision Medicine Research. J Pers Med. 2016 Jun 9;6(2):E17

24. Sather S. The Essential Guide To Electronic Informed Consent. Signant Health (eBook); 2016

25. Wilbanks J. Design Issues in E-Consent. J Law Med Ethics. 2018 03;46(1):110-8

26. Moore S, Tassé AM, Thorogood A, Winship I, Zawati M, Doerr M. Consent Processes for Mobile App Mediated Research: Systematic Review. JMIR Mhealth Uhealth. 2017 Aug 30;5(8):e126

27. Simon CM, Klein DW, Schartz HA. Traditional and electronic informed consent for biobanking: a survey of U.S. biobanks. Biopreserv Biobank. 2014 Dec;12(6):423-9

28. Chen C, Turner SP, Sholle ET, Brown SW, Blau VLI, Brouwer JP, et al. Evaluation of a REDCap-based Workflow for Supporting Federal Guidance for Electronic Informed Consent. AMIA Jt Summits Transl Sci Proc. 2019;2019:163-72

29. Simon CM, Schartz HA, Rosenthal GE, Eisenstein EL, Klein DW. Perspectives on Electronic Informed Consent From Patients Underrepresented in Research in the United States: A Focus Group Study. J Empir Res Hum Res Ethics. 2018 10;13(4):338-48

30. Paterick, Timothy J., Barbara B. Paterick, and Timothy E. Paterick. Expanding electronic transmissions in the practice of medicine and the role of electronic informed consent. Journal of Patient Safety 4.4 (2008): 217-220

31. Lunt H, Connor S, Skinner H, Brogden G. Electronic informed consent: the need to redesign the consent process for the digital age. Intern Med J. 2019 07;49(7):923-9

32. Kang, Hee Sun, and Sun-Hee Kim. Experiences of Patients and Nurses Regarding the Use of Electronic Informed Consent. Session presented on Sunday, July 27, 2014, 25th International Nursing Research Congress, Hong Kong. Available from: https://sigma.nursingrepository.org/handle/10755/335312?show=full (Accessed 20th February 2020
